# The place of digital triage in a complex healthcare system: An interview study with key stakeholders in Australia's national provider

**DOI:** 10.1177/20552076231181201

**Published:** 2023-06-23

**Authors:** Kate Churruca, Louise A Ellis, Catherine Pope, Jennifer MacLellan, Yvonne Zurynski, Jeffrey Braithwaite

**Affiliations:** 1 Australian Institute of Health Innovation, 7788 Macquarie University, Sydney, Australia; 2 Nuffield Department of Primary Care Health Sciences, 6396 University of Oxford, Oxford, UK

**Keywords:** Health systems, digital triage, telephone triage, Healthdirect helpline, stakeholders’ perspectives

## Abstract

**Background:**

Digital triage tools such as telephone advice and online symptom checkers are now commonplace in health systems internationally. Research has focused on consumers’ adherence to advice, health outcomes, satisfaction, and the degree to which these services manage demand for general practice or emergency departments. Such studies have had mixed findings, leaving equivocal the role of these services in healthcare.

**Objective:**

We examined stakeholders’ perspectives on Healthdirect, Australia's national digital triage provider, focusing on its role in the health system, and barriers to operation, in the context of the COVID-19 pandemic.

**Methods:**

Key stakeholders took part in semi-structured interviews conducted online in the third quarter of 2021. Transcripts were coded and thematically analysed.

**Results:**

Participants (n  =  41) were Healthdirect staff (n  =  13), employees of Primary Health Networks (PHNs; n  =  12), clinicians (n  =  9), shareholder representatives (n  =  4), consumer representatives (n  =  2) and other policymakers (n  =  1). Eight themes emerged from the analysis: (1) information and guidance in navigating the system, (2) efficiency through appropriate care, (3) value for consumers? (4) the difficulties in triage at a distance, (5) competition and the unfulfilled promise of integration, (6) challenges in promoting Healthdirect, (7) monitoring and evaluating digital triage services and (8) rapid change, challenge and opportunity from COVID-19.

**Conclusion:**

Stakeholders varied in their views of the purpose of Healthdirect's digital triage services. They identified challenges in lack of integration, competition, and the limited public profile of the services, issues largely reflective of the complexity of the policy and health system landscape. There was acknowledgement of the value of the services during the COVID-19 pandemic, and an expectation of them realising greater potential in the wake of the rapid uptake of telehealth.

## Introduction

Telephone health triage and advice services have been introduced in many countries such as Sweden, Australia, Saudi Arabia and the United Kingdom over the past two decades.^1–3^ Such services are staffed by clinicians^1^ or trained nonclinical call handlers^4^ utilising an evidence-based computer decision support system (CDSS) to elicit information on callers’ symptoms and advise on the level of care for their needs. More recently, online symptom checkers have become widely available and can provide a self-paced supplement or replacement for telephone triage.^5^ For example, Healthdirect in Australia, NHS 111 online in the UK and Sweden's 1177 Vårdguiden operate digital triage services that include both online symptom checkers and helplines.

### Background

At a health system level, reasons for implementing digital triage services typically include a desire to manage demand by reducing—for those advised self-care—or diverting away from emergency, to improve access in the afterhours period or to realise other promises associated with technological efficiency (e.g. replacing staff or relying on less expensive staff).^2,6,7^ However, research to support digital triage services reducing health service demand is equivocal; some studies have reported a reduction in service use, while others have found no change.^8,9^

There are large discrepancies among studies on the accuracy of advice provided by digital triage services. Online symptom checkers have been estimated to recommend the appropriate level of care only about half the time.^5^ They are regarded by many as overly ‘risk averse’, advising users to seek healthcare where self-care is appropriate.^5,10^ One systematic review of telephone triage services found that the median for appropriate triage advice was 75%, with evidence of under-referral in the range of 0.2–50% of calls.^11^

The health benefits of these online and telephone platforms are realised only if patients and users follow the advice given, and estimates of this vary across studies and by type of advice provided;^8,9,11^ for example, among users of the Healthdirect helpline that were 45 years or older, compliance was 77.5% for self-care and 68.6% for ‘attend ED immediately’, while 7.0% of callers self-referred to the emergency department (ED) within 24 hours.^12^ For NHS 111, users are compliant only about half the time.^13^ Generally, users of digital triage services feel reassured and satisfied with the experience, although this also varies by the type of service and type of advice.^2,5,9–11^

By virtue of their span across the health system and their relative ease of access through the internet or phone, digital triage services have a potentially wide array of influence from consumers to primary care to hospitals and specialist clinicians. However, since their implementation internationally, no study has investigated the perspectives of key stakeholders such as policymakers, health system partners and consumers on their role in the health system. There is cause to expect diverse, even polarised views among stakeholders, though; in a case study of the telephone NHS 111 service, Pope et al.^14^ noted representations varying from ‘a high-quality service in the face of high demand’ to ‘the source of an increasing and inappropriate burden on over stretched NHS ambulance and emergency care services’ (p. 1) in reviews and media reports.

### The present study

Understanding the perspectives of key stakeholders may highlight systemic benefits, opportunities and challenges to digital triage services that are not recognised in the literature evaluating user experiences, clinical utility and outcomes. Moreover, the pandemic has underscored the potential value of these services, both in providing remote access to evidence-based health advice—thereby facilitating social distancing—and in managing some of the additional demand for healthcare wrought by COVID-19.^15,16^ With these issues in mind, the present study aimed to examine stakeholders’ perspectives on a national digital triage service and its role in the health system, in the context of the COVID-19 pandemic. The research questions were:

1. How do the digital triage services (online and telephone) work together and what role do they serve in the health system?
2. What are stakeholders’ perceptions of the impacts of digital triage on the healthcare system? What are the perceived barriers?
3. How has the COVID-19 pandemic affected the landscape of digital triage services?

## Method

This was a qualitative study involving semi-structured interviews with key stakeholders in a national digital triage service—Healthdirect—with thematic analysis of their accounts. The research was conducted in conjunction with a large program of work looking at online triage services in England and comparing them with Australian services.^17^ We took a post-positive theoretical stance, which assumes that there is an objective social reality but that people's perceptions of it are inherently subjective.^18,19^ The study followed the COnsolidated criteria for REporting Qualitative research (COREQ) checklist.^20^

### Context and setting

To understand the relevant stakeholders for Healthdirect, and the different issues they described in digital triage, a brief overview of the service and system it is situated within is required. The Australian healthcare system is complex and crowded with public and private actors operating at local, regional, state and national levels. Although there is some degree of free or heavily subsidised healthcare, general practitioners (GPs) mostly operate as businesses and have discretion in setting their own fees. While for the most part they are reimbursed via Medicare (bulk-billing), many will add a fee on top; although 86% of GP services were bulk-billed nationally in 2018–19, only 66% of patients had *all* services bulk-billed.^21^ GPs are also unequally distributed across Australia, with a far greater concentration in cities leading to potential access issues for patients in rural, regional and remote areas.^21^

General practice and community health is supported by 31 Primary Health Networks (PHNs). These federally managed initiatives conduct needs assessments and commission services for their local populations, including afterhours services, and aim to improve coordination between hospitals and primary care. Coordination is challenging partly because public hospitals in Australia are managed at a state level through shared funding arrangements with the Federal government. There is also weak integration between hospital and primary care systems, and poor information technology interoperability.

Healthdirect was established in 2006 under an agreement between state and federal governments to centralise their health advice phone lines.^22^ Most states and territories (n  =  6/8, 75%) signed on, except Queensland and Victoria. Since 2021, Victoria has accepted the helpline but continues to brand it under its own NURSE-ON-CALL name. These governments, with the exception of Queensland, fund the service and are therefore called its ‘shareholders’. The helpline is staffed by registered nurses who conduct triaging using a CDSS. Healthdirect added an online presence in 2009 to provide ‘safe, local health information’, and the After Hours GP Helpline was implemented as a call-back service to the nurse-run Healthdirect line in 2011. In 2014, Healthdirect launched its web-based Symptom Checker, which became available on its new phone app a year later.

Across these triage services, users are provided with information about their symptoms and how to manage them and a disposition, which is a recommended course of action based on symptoms and circumstances. Dispositions typically fall into one of three categories: self-manage at home, see a GP (within hours or days) or present to an ED. Triage is supported by the Healthdirect-managed National Health Services Directory (NHSD), which collates location and service information on all Australian healthcare providers, allowing a triaging nurse or a consumer using the Symptom Checker to identify *where* specifically they can go to access appropriate services for their health issue. Users of the services are also able to receive a personalised summary of their triage advice and care information as a bespoke weblink that is sent to their phone via SMS.

### Participants and recruitment

We used purposive sampling, seeking perspectives from stakeholders involved in, interacting with, or affected by, Healthdirect's triaging system. An initial list of relevant stakeholders included key Healthdirect operational staff and senior leadership, government shareholder representatives, GPs and ED clinicians whose patients used the service and consumer representatives. This list was refined, with additional stakeholder groups targeted through the course of data collection. For example, a couple of Healthdirect staff highlighted the importance of the service's relationship with PHNs, as the latter had a remit to improve care coordination and afterhours access, so these were added. Healthdirect operational staff included those who oversaw staffing, the accuracy of digital content, infrastructure, marketing and health system partnerships; they did not include current call handlers because the management of staff for the helpline was provided under contract by a separate provider.

Recruitment was conducted in four ways: (1) facilitated by Healthdirect leadership; (2) contacting organisations (e.g. PHNs, Royal Australian College of General Practitioners) to request that they distribute information about the study to relevant members/employees; (3) reaching out to suitable contacts of the research team to gauge their interest in taking part; and (4) snowballing, where some participants forwarded details to other potential stakeholders. In all instances, a personalised email with standard participant information and a consent form attached was used to introduce the study. The email explained that the study was about stakeholders’ perspectives of Healthdirect and noted why the individual was considered a stakeholder (e.g. for an ED nurse: ‘I am interested in speaking with people who work in ED because presenting patients may have initially contacted Healthdirect for triaging advice’). We anticipated that a sample size of 30 to 50 would be sufficient to reach data saturation, based on our previous work, and proceeded with recruitment until this was achieved. No individual who expressed interest in taking part dropped out.

### Data collection

Interviews were conducted by KC (PhD) or LAE (PhD); both women are experienced health services researchers and qualitative interviewers. While neither had previously researched digital triage services, other team members had,^14,23^ and some had used the helpline in a limited capacity to seek advice for themselves or their children and found the experience satisfactory. Most interviewees had no prior relationship with the researchers, though a couple were professional acquaintances from other research projects.

A semi-structured interview schedule was developed asking participants about their current role and organisation, level of knowledge and involvement with Healthdirect, views on how its triage services are used and by whom, the impact of Healthdirect on the broader health system, and challenges faced with Healthdirect. It was workshopped amongst the research team, but no formal pilot testing was undertaken.

Interviews took place from July to August 2021 and were conducted over Zoom or Microsoft Teams depending on participant preference. In three cases, interviews were conducted simultaneously with multiple participants who were representatives of the same organisation; they requested this format for efficiency and completeness. We did not explicitly ask about COVID-19 during interviews, though it was a recurrent topic. Interviews were audio-recorded using both the teleconference platform inbuilt recording capability and Otter auto-transcription technology.^24^ Interviews lasted approximately 30 to 45 minutes in duration.

### Data analysis

Interviews were transcribed using the auto-transcription service Otter, and then de-identified and integrity checked by a research assistant or KC. De-identification was conducted at both an individual and an organisational level, such that participating PHNs or policy organisations would not be identifiable. Transcripts were then imported into NVivo for coding. Because of time constraints, participants were not provided with an opportunity to review their transcripts.

Aligned with our post-positivist stance, we used thematic analysis to focus on stakeholders’ meanings and experiences of Healthdirect ‘to gain insights into the external reality, thereby supporting the development of conjectural knowledge about reality’ (p.847).^25^ A draft coding framework was developed inductively by KC and LAE after approximately two-thirds of interviews were complete. This framework focused on the process and pathways of digital triage, its purpose(s), challenges and contextual issues, as well as double coding for the specific services (e.g. helpline, Symptom Checker) being referred to. Several revisions to the framework were made throughout the coding process to better capture, for example, the context of Healthdirect's triage system (e.g. demand on EDs, access to services in rural-remote Australia). Coding was conducted by KC and coded categories were read through and confirmed by LAE. Broader themes were developed by grouping related codes together to address the research questions. These themes were reviewed and refined in the process of defining them.^26^ They are summarised below with exemplary quotes from participants, with their ID and main stakeholder position.

### Ethical issues

The ethical conduct of this research was approved by a Human Research Ethics Committee at Macquarie University (reference number 52021985728536) with all participants providing voluntary informed consent to take part.

## Results

Thirty-seven interviews were conducted with 41 participants. The primary role or perspective represented for each participant is summarised in Table 1. However, this categorisation did not capture the multiple vantage points of some participants. For example, a few interviewees working at Healthdirect and in PHNs had a clinical background, and numerous participants from policy/shareholder-representative roles had used the digital triage services in their personal lives.

**Table 1.** Primary role of participating stakeholders.

Stakeholder group	N	%
Healthdirect staff^a^	13	31.7
PHN Representatives^b^	12	29.3
Clinicians^c^	9	22.0
Shareholder Representatives and other policymakers	5	12.2
Consumer Representatives	2	4.9
Total	41	100

^a^ Covering senior leadership and operational staff.

^b^ Across eight PHNs.

^c^ GPs, ED doctors and nurses, and mental health providers working in the health system with which Healthdirect interfaces.

**Figure 1.**
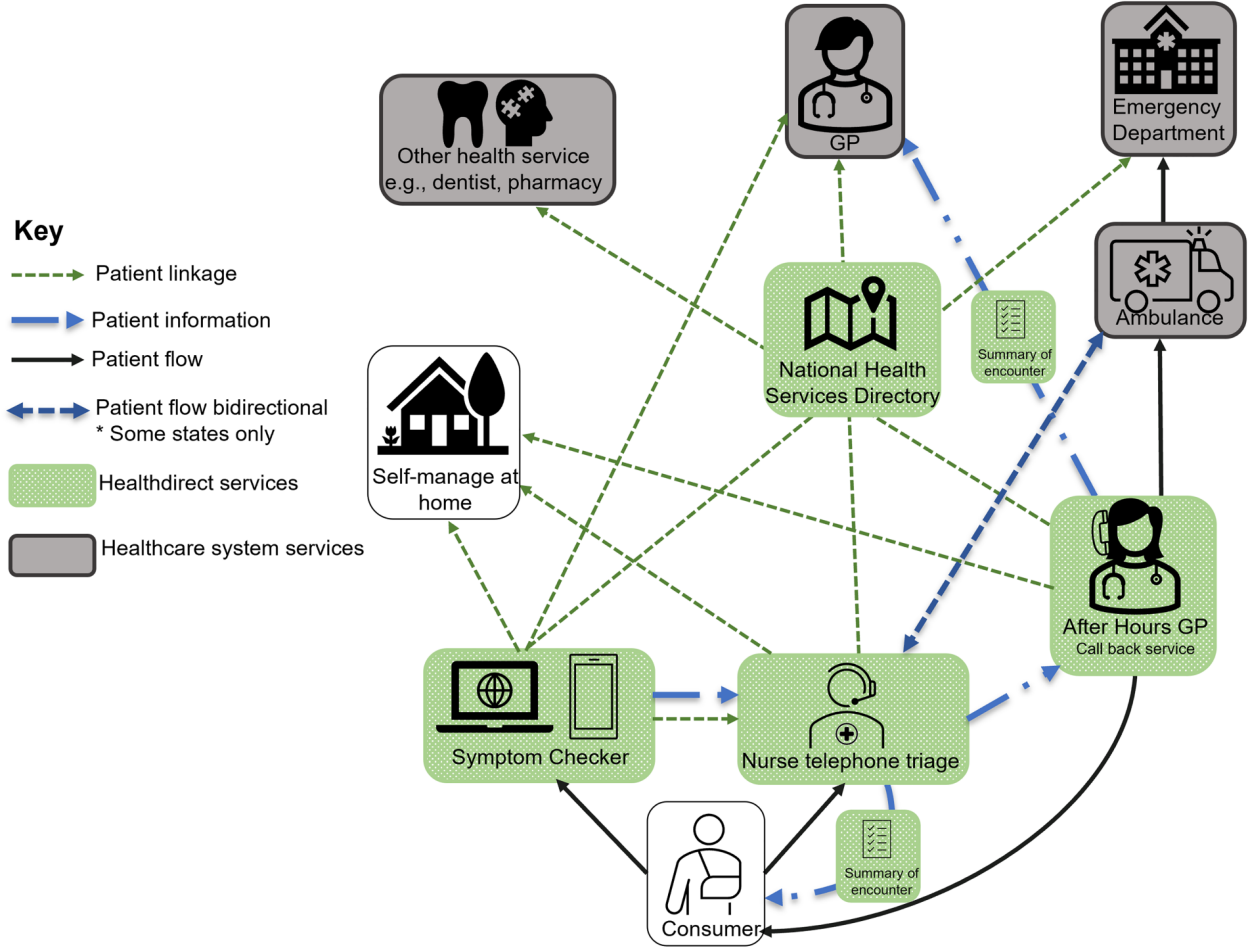
Core components of Healthdirect's triaging service and their interactions with other parts of the Australian healthcare system.

During interviews, we refined our understanding of the different components of Healthdirect's digital triage services and their interface with the wider health system. A simplified schematic of this interaction is displayed in Figure 1. We identified eight themes from the analysis of participants’ interviews to address our research questions: (1) information and guidance in navigating the system; (2) efficiency through appropriate care, (3) value for consumers? (4) the difficulties in triage at a distance, (5) competition and the unfulfilled promise of integration, (6) challenges in promoting Healthdirect, (7) monitoring and evaluating digital triage services and (8) rapid change, challenge and opportunity from COVID-19. We now describe each theme with exemplar extracts of data.

### Information and guidance in navigating the system

According to Healthdirect staff, shareholders and policymakers, and PHN and consumer representatives, Healthdirect was an important resource for nationally consistent, evidence-based health information and advice: ‘the goal would be to be a trusted information source, an impartial, unbiased information source for the Australian population’ (Policymaker 1). This was particularly important within the highly complex, crowded and constantly changing environments of both Australia's healthcare system and the digital health landscape.

Moreover, while Healthdirect was not required for a user to access primary or emergency care, the service was seen as a reliable ‘entry point’ or ‘gateway’ into this system, one that helped to ‘navigate’ complexity. It also sometimes acted as a waypoint, with one Healthdirect staff member explaining how one of the most common categories of helpline caller was patients seeking clarification about care or treatment initiated in the health system: ‘effectively people who had been to see their GP and been diagnosed and had got the medication… but then were calling Healthdirect, to check something to do with their previous visit to the GP’ (Healthdirect staff 9).

### Efficiency through appropriate care

Some shareholder representatives and Healthdirect staff acknowledged that the helpline had been introduced to take pressure off other parts of the system, especially EDs: ‘the service was set up to help alleviate pressure on the health system in Australia, as well as ensuring people get access to the right care, at the right time, in the right place…. part of the journey along the way it evolved over time, and we’ve built on the additional digital tools [Symptom Checker] to help alleviate pressure on the call handling and manage call volume’ (Shareholder Rep. 2). This highlighted the evolving nature of the service and the somewhat ironic issue of demand management requiring its own demand management over time.

Participants varied in whether they viewed digital triage as achieving this goal. One former staff member (Healthdirect staff 1) said ‘it didn’t reduce ED admissions… [but] it might have achieved more appropriate presentation to EDs elsewhere’, while a current senior staff member (Healthdirect staff 5) said ‘it's about 70 or 80%, [of callers] who said that [they’d otherwise] go to ED actually get a lower disposition. So, I think we are channelling a lot of people away from ED’. Some questioned whether ED diversion was a fair or appropriate goal for digital triage: ‘efficacy in terms of keeping people out of hospital is not tested over primary care all the time and it's, it's an interesting lens to apply just to the Healthdirect service… there is an over emphasis… the emphasis should really be on right care, right time’ (Healthdirect staff 3). Many participants preferred the focus of ‘right care, right time’ over demand management, as these quotes suggest. One Healthdirect staff member (10) indicated this would result in greater ‘efficiency’ and ‘effectiveness’ of the wider healthcare system too, and a PHN Representative (PHN Rep. 7) mentioned adopting the Symptom Checker in their community for this reason: ‘…it's about finding the right service … We think it's a good way for people then to understand what the different services are so… resources are allocated appropriately within the health system and meet the patient's needs.’

### Value for consumers?

A range of benefits for users of the digital triage services were mentioned by Healthdirect staff and consumer representatives. The helpline was singled out for providing support and emotional reassurance during a stressful time: ‘assurance or direction… particularly if there is the opportunity for that person to move a little bit off script to provide that’ (Consumer Rep. 1). Both online and phone triage services were also described as fast, efficient and easily accessible, which supported equity of access in a geographically large country: ‘There's a massive equity lens that you apply … telephone ownership even in rural and remote is very high so it provides… a base service to everyone’ (Healthdirect staff 3). Some Healthdirect staff also suggested that the service could improve health literacy by educating people, for example, about appropriate medication use or how to manage symptoms at home.

However, clinical stakeholders were far more sceptical of these potential benefits. One ED nurse explained that ‘the demographic that I serve where a lot of people have English as a second language… health literacy is quite poor… they’re all chronically unwell… I just don’t know that these people would be willing to [use Healthdirect]’ (Clinician 5). A GP suggested the service added to, rather than alleviated her patients’ distress, sending them out to seek care after hours and contributing to a view that all illnesses required immediate consultation. Another nurse spoke of occasions where patients had been referred to ED by Healthdirect, with the designation that they required emergency care giving them a false expectation of the urgency of their condition: ‘you are desperate with a kid… you’ve never seen them this sick… you get a little bit of clarity, that is “go to ED”… then that's very invalidating when… a triage nurse [in ED] is absolutely brutal to you and tells you you're gonna wait there for eight hours’ (Clinician 9). Despite reservations, most acknowledged their skewed impression of the service, as they only saw the people referred on to care, and were not made aware of the bigger picture, the ‘denominator’ of users (Clinician 3).

Finally, although shareholders and PHN representatives viewed digital triage as having value for the people in the communities they served, some noted that the national service model led to a lack of understanding of the local context, which could sometimes result in unhelpful advice for service users: ‘Healthdirect is … quite underutilized [in Western Australia] … a lot of the time … referral to the nearest ED could be 300 kilometres away. So there's that lack of context and understanding about just how far away the next service is from Healthdirect's perspective’ (PHN Rep. 3).

### The difficulties in triage at a distance

Related to the scepticism among clinicians described above, there were conflicting views about whether digital triage advice sent users to a higher acuity service than their condition warranted: ‘all roads lead to “go to the ED”’ (Clinician 9). One shareholder cited longitudinal data that suggested an increase in the proportion of people being given the advice to go to ED: ‘But I can see no data that says people are sicker’ (Shareholder Rep. 4).

Although Healthdirect staff reiterated the evidence-based and appropriate nature of their advice, there were a few examples where a user would be directed to a higher level of care such as if they were advised to see a GP in the afterhours period, where none was open: ‘in the event that they need to be seen by a health professional, the emergency department ends up being where they land’ (Clinician 2). Moreover, Healthdirect staff conceded that in their own ways the Symptom Checker and the helpline both ‘err on the side of caution’ (Healthdirect staff 6). There was acknowledgement among staff and clinicians—many of whom had provided telehealth during the pandemic—of the challenges in triaging without a physical examination or often even seeing a patient. Indeed, a few clinical stakeholders said that the advice Healthdirect provided was appropriate given these limitations and noted how all healthcare was similarly concerned with minimising risk, with one ED doctor saying ‘it's inevitable that… the risk you’re prepared to accept is going to be a lot less. You can’t see them, and you can’t call them up. Once they hang the phone up, you don’t know what's going to happen… I think it's appropriate, they’re doing exactly what I would do if I was put in that situation’ (Clinician 3).

### Competition and the unfulfilled promise of integration

Interviewees from Healthdirect and government shareholders noted issues in the integration of its different digital triage services, particularly the ‘clunky’ transition if a user was required to shift from the Symptom Checker to the helpline. There was also a perception that Healthdirect did not integrate well with the wider health system, although a few specific examples to the contrary were highlighted (e.g. users of After Hours GP could have a summary sent to their regular GP; secondary triage for ambulance services). As Shareholder Representative 4 said: ‘one of the problems with Healthdirect is its lack of integration with every everything and everywhere’. Some participants contextualised this by noting that poor integration was symptomatic of healthcare more generally: ‘communication between different providers can be very poor… if they’ve being say treated in emergency, they … might simply be “yes you’re free to go now”… the information about what happened is not necessarily communicated to their primary carer’ (Shareholder Rep. 1). Indeed, a couple of stakeholders working or affiliated with Healthdirect suggested that there was an opportunity for the organisation to contribute to integration by virtue of its national profile and digital acumen.

Further to integration issues, PHN representatives mentioned commissioning a range of services that overlapped with, or even duplicated parts of Healthdirect's offering; these other services were described as having a wider scope or additional capabilities in treatment (e.g. e-prescribing). One Healthdirect staff member (10) acknowledged this ‘longer term challenge… (of) our participation in a growing digital or virtual care industry … we’re government funded organisation, I don’t want to be competing’. Nevertheless, there were sometimes suggestions that traditional healthcare services also competed with Healthdirect, either directly for patients or indirectly for finite funding. For example, an ED doctor (Clinician 3) alluded to activity-based funding, explaining how the funding of his department broadly came from the ‘number of presentations’ and that if Healthdirect had any effect on the system, it would be in reducing presentations for people where there was ‘nothing important’ wrong with them—as these people were easier to care for, they balanced the additional resources required to care for frail, medically complex patients. Although recognising that ‘there's interest groups that get a bit threatened if… it looks like somebody is coming into their area’, another Healthdirect staff member (5) emphasised that they designed their services to ‘complement, and never ever (be) in competition with them’.

### Challenges in promoting Healthdirect

One of the major challenges to Healthdirect's digital triage services within this complex and competitive environment was a lack of public awareness among consumers and healthcare providers: ‘I bet if I rang or contacted 50 people around the country … and said, ‘Have you used Healthdirect?’ I can guarantee I would get the ‘huh, what's that?’… I don’t think we actually sell it as a resource’ (Consumer Rep. 1). Stakeholders noted that there was limited ability to promote the helpline because government shareholders placed restrictions on the number of calls it could receive. Any promotion might increase demand beyond these call caps—a ‘catch 22’ (Healthdirect staff 2). While the online Symptom Checker did not require demand management, it was challenging to promote because the generalist nature of its content made it difficult to find an audience: ‘it is every single health issue that you ever heard of, promoting it to every single Australian’ (Healthdirect staff 9).

Some staff suggested that Healthdirect was not well branded, and that the non-specific name of the service and long helpline phone number made it difficult to identify. The use of Healthdirect for both the triage helpline and the larger organisation also contributed to confusion among PHNs and healthcare providers about its scope of practice because the organisation offered a range of other digital health solutions (e.g. video call consultation platform): ‘the brand is a familiar name … to staff in (general) practices but again, the scope of what it can do, is not necessarily, no’ (PHN Rep. 9). Sometimes interviewees juxtaposed these issues with England's NHS 111, where there was a seemingly more integrated and clearly branded health system that had been used in naming the triage service, which itself was marketed to clearly differentiate its role in urgent care: ‘it's just been it's just been sold perfectly in the UK… There's a really clear differentiation between, you know, ED and GP, what it's for’ (PHN Rep. 2).

### Monitoring and evaluating digital triage services

Healthdirect collected and used data extensively to monitor the quality of its services and user satisfaction, solicit feedback for improvement and plan for future utilisation. However, they did not generally collect data suitable for tracking patient journeys, such as via the use of Medicare information, meaning there was a lack of knowledge on where people went after using the service. Data linkage studies had been conducted in the past to evaluate patient adherence and health outcomes, but these were costly, ad hoc and had focused on the helpline only, whereas far more people used Healthdirect's online content than the phone service: ‘We don’t know how people use digital health information to impact their behaviours… we don’t know what proportion of their behaviour can be explained by the use of digital health information’ (Shareholder Rep. 4). Inability to follow patients through the health system, especially after accessing the Symptom Checker, limited understanding of the outcomes of using digital triage and diminished Healthdirect's ability to identify and demonstrate its value to funders: ‘how can we track the patient journey so that we can provide the insights to the fund providers about where the consumer has ended up on that journey?’ (Healthdirect staff 8).

### Rapid change, challenge and opportunity from COVID-19

The pandemic created a range of challenges and opportunities with Healthdirect, an important part of the national response to COVID-19. Staff highlighted how the organisation and its services had always been flexibly arranged to permit rapid scaling up during emergencies. For example, a separate helpline was created and independently staffed to provide guidance on COVID-19. However, there was still increased demand for the standard helpline: ‘people have questions about “is it safe for me to get a vaccine?” and they’re still ringing the Healthdirect line… they don’t ring the National Coronavirus Helpline’. The same Healthdirect staff member (10) went on to explain how this demand was challenging because of a ‘resource crunch’ in the lack of ‘availability of nursing staff’. Nurses were in high demand for various roles in the pandemic response (e.g. administering vaccines), and without the typical flow of skilled health professionals from overseas, due to government border restrictions. This led Healthdirect to reconfigure the management of its telephone service workforce moving forward, making it more resilient to such wide variation in demand by shifting from a single provider to a distributed model.

Healthdirect also had to establish an online team to review, synthesise, simplify and disseminate evolving health information, recommendations and restrictions: ‘There's a whole separate team that deals with COVID… they made literally thousands of changes on the fly… seven day a week shift, seven to 10pm because it's changing so rapidly’ (Healthdirect staff 6). This team created numerous interactive tools similar to the Symptom Checker, specifically for COVID; for example, a vaccine eligibility checker which, after confirming eligibility, could take a user directly to a live portal to book a vaccine in their area. This functionality was made possible by agreements with online healthcare booking platforms, and although it came about because of COVID, it extended beyond this to booking regular visits to a GP.

The rapid adoption of telehealth across the health system during the pandemic was seen by some as providing Healthdirect with an opportunity to increase its national profile. For example, one GP who had worked with Healthdirect said ‘the COVID situation has definitely helped in identifying their role… When you’re watching [Healthdirect] now be the place where you check if you’re eligible for vaccines, wow, could they have not been doing stuff like that before… it took this. They’re the Symptom Checker, they’re the ones that tell you if you should be getting tested’ (Clinician 4). Moreover, some Healthdirect staff suggested the pandemic as enhancing the acceptability of digital health tools and services among society more broadly, something they might leverage in the future: ‘It's really accelerated people's knowledge of health and their own health and what is credible health information. And also that idea that digitising or seeking health information online and going through tools and checkers and connecting and making a booking with a GP online and having that booking via a video’ (Healthdirect staff 9).

## Discussion

This study examined key stakeholders’ perspectives of a national digital triage service and its role in the health system, taking Australia's Healthdirect as our case. We found that digital triage services, composed of both phone and online components, are seen as having an important place in supporting consumers to navigate through the health system to the appropriate care for their needs. These services face challenges in providing advice while managing the risks inherent in triaging at a distance, in funding and promotion of their services, as well as in providing a complementary and demonstrably useful service within a complex, fragmented and poorly integrated health system.

Fragmentation and duplication of effort are common issues in Australia's healthcare system, partly due to shared and overlapping responsibilities of state and federal governments, the mix of public and private providers, regionalisation and lack of integration of services.^27^ The fragmentation was particularly evident in our study in the variability of attitudes to, and knowledge and use of Healthdirect services by representatives of the federally funded, locally managed PHNs. Echoing our findings, Freeman et al.^28^ identified heterogeneity in the degree of collaboration between Australian PHNs and an array of state and territory actors (e.g. state Department of Health, Local health district). In this regard, the mandated role of PHNs in commissioning services has been suggested to perpetuate competition over collaboration,^29,30^ which was a theme in the present study.

Participants who worked as clinicians in the health system were uncertain of the value of Healthdirect for their patients, practices or the broader health system. By comparison, Kujala et al.^31,32^ found that Finnish health professionals were positive about their patients using a symptom checker for a range of common health issues; however, this service was part of a broader patient portal that supported self-management and provided symptom information to providers, something our participants were receptive to but had never encountered with Healthdirect. Indeed, clinicians in our study had a greater awareness of the telephone service than the Symptom Checker, despite the latter being more widely used.^33^ Comments from Healthdirect staff during interviews shed some light on this, referring to data showing that people using the helpline typically had higher acuity conditions, which might require referral. In many instances, while clinicians were cognizant of the challenges in remote triage, they viewed the national provider variably, as either too cautious or else an unnecessary intermediary to a foregone endpoint.

Many participants, including Healthdirect staff, consumer representatives, policymakers and some PHNs, saw the organisation as having a pivotal role during the COVID-19 pandemic, as it rapidly scaled up and adapted its online and telephone services to meet increased demand for health advice and access to vaccines. A time series analysis of the use of New Zealand helplines during the pandemic found peaks coinciding with lockdowns or spikes in infection, but also that demand ‘stablized at a new higher level' (p. 1) compared with that before COVID.^34^ This would seem to support the idea of widespread increased acceptance and use of these types of remote services that could be leveraged into the future. However, Duckett^35^ has argued that in light of the pandemic, Australia requires a single national health phone line that is widely recognised and integrated with the primary health care system. While Healthdirect goes a long way toward fulfilling these needs, our findings highlight issues in its promotion and public awareness, its differential arrangements and branding between states, and its lack of integration with the wider healthcare system that suggest that it is not currently fit for this purpose.

### Implications

In some sense, a national digital triage service such as Healthdirect is a paradox; its array of influence is large—from primary, hospital and community care—while its impact on the system is limited, or at least difficult to identify, or measure. Similarly, a before and after study by Knowles et al.^36^ found that implementation of the NHS 111 phone service did not affect the population's perceptions of urgent care, likely because of the ‘small amount of NHS 111 activity in a large emergency and urgent care system’ (p. 1). Although Healthdirect does not appear to be a well-recognised service, its perceived potential, particularly in a post-COVID era, is considerable. Many of the challenges it faces are a reflection of the complex healthcare system in which it is situated,^37^ or related to the politics of its funding, neither of which are easily addressed. We have identified a few areas that could, however, be targeted in the future to the improve understanding and use of digital triage; these areas are summarised in Table 2.

**Table 2.** Summary of select implications drawn from findings.

Recommendation	Implication type	Details
Learn from the international comparators	International research	Other high-performing health systems such as Sweden and England have implemented similar digital triage services. Comparison of the challenges and differences in their configuration might lead to innovation and ideas for improvement.
Evaluate other potential consumer benefits of using digital triage services	National research	Stakeholders mentioned broader outcomes of using digital triage in empowering consumers and supporting their health literacy. These potential benefits, as well as other factors like why consumers use the service, should be rigorously evaluated.
Actively disseminate information on the service to PHNs, general practices and EDs	Policy	These stakeholders working in the health system may play a role in promoting the appropriate use of the triage services to patients and the public but in this study frequently reported being unaware of Healthdirect's features (e.g. challenges in promoting Healthdirect) or unconvinced by its potential to support consumers (i.e. value for consumers?). Providing information on scope, utilisation and referral rates would support their understanding.
Integrate online and telephone services	Service improvement	Transitions between Healthdirect's symptom checker and its telephone helpline were not seamless; consumers had to re-provide symptom details. Improving integration would lead to a more seamless user experience, expedite assessments, and allow for better patient tracking across services.
Support further integration of the triage services with the health system	System improvement	Improving the integration of digital triage services with the health system would allow a greater understanding of patient journeys and how Healthdirect influences consumer behaviour, both for online and helpline.

### Strengths and limitations

Our study provides the first comprehensive analysis of multi-stakeholder views of the benefits and challenges faced by large-scale digital triage services. We followed recommended guidelines for the collection, analysis and reporting of qualitative data; however, such broad research questions and diverse participants made it difficult to do justice to some of the complexity and nuances in their individual accounts. Our multipronged recruitment strategy yielded representation from one-third of Australian PHNs, senior leadership and operational staff at Healthdirect, numerous high-level policymakers and experienced clinicians working in the health system with varying degrees of formal and informal involvement with the triage services. Although it is not possible to make claims about the generalisability of these viewpoints, they come from highly influential and knowledgeable actors with extensive experience in digital triage and healthcare delivery. One limitation is the small number of consumer representatives among our interviewees, although many participants had been *consumers* of Healthdirect. This is unfortunate given the numerous claims made about the value of Healthdirect that privileged consumers; this provides an opportunity for further research (Table 2).

## Conclusions

Research on digital triage has largely focused on experiences and consequences for individual users and call handlers. Our study has taken a step back to consider the role of a national digital triage service in a complex, fragmented, but nevertheless high-performing health system. Healthdirect occupies a liminal space in Australia's healthcare system. Despite interacting with numerous services, a goal to complement the care they deliver, and the potential offered by digital solutions, it does not join them up, instead, acts as a separate or add-on feature. Its potential to streamline patient journeys and demonstrate improved clinical outcomes is curtailed by the lack of health system integration, particularly across digital platforms. Nevertheless, there may be other benefits to consumers in giving them direct access to evidence-based advice and information, and certainly, there is an opportunity to improve these services following the COVID-19 pandemic.

## Supplemental Material

sj-docx-1-dhj-10.1177_20552076231181201 - Supplemental material for The place of digital triage in a complex healthcare system: An interview study with key stakeholders in Australia's national provider Click here for additional data file. Supplemental material, sj-docx-1-dhj-10.1177_20552076231181201 for The place of digital triage in a complex healthcare system: An interview study with key stakeholders in Australia's national provider by Kate Churruca, Louise A Ellis, Catherine Pope, Jennifer MacLellan, Yvonne Zurynski and Jeffrey Braithwaite in DIGITAL HEALTH

## References

[1] Ernesäter A Engström M Winblad U , et al. A comparison of calls subjected to a malpractice claim versus ‘normal calls’ within the Swedish healthcare direct: a case–control study. BMJ Open 2014; 4: e005961. 10.1136/bmjopen-2014-005961 PMC4187455 25280808

[2] Bunn F Byrne G Kendall S . The effects of telephone consultation and triage on healthcare use and patient satisfaction: a systematic review. 2005; 55: 956–961. PMC1570504 16378566

[3] Alfaleh A Alkattan A Alageel A , et al. The role of telemedicine services in changing users’ intentions for presenting to the emergency departments in Saudi Arabia. Digital Health 2022; 8: 20552076221091358. 35694122 10.1177/20552076221091358 PMC9185009

[4] Anderson A Roland M . Potential for advice from doctors to reduce the number of patients referred to emergency departments by NHS 111 call handlers: observational study. BMJ Open 2015; 5: e009444. 10.1136/bmjopen-2015-009444 PMC4663401 26614624

[5] Semigran HL Linder JA Gidengil C , et al. Evaluation of symptom checkers for self diagnosis and triage: audit study. Br Med J 2015; 351: h3480. 26157077 10.1136/bmj.h3480 PMC4496786

[6] Nagree Y Cameron P Gosbell A , et al. Telephone triage is not the answer to ED overcrowding. Australia: Blackwell Publishing Asia Melbourne, 2012, pp. 123–126. 10.1111/j.1742-6723.2012.01547.x 22487660

[7] Grol R Giesen P van Uden C . After-hours care in the United Kingdom, Denmark, and the Netherlands: new models. Health Aff 2006; 25: 1733–1737. 10.1377/hlthaff.25.6.1733 17102200

[8] Lake R Georgiou A Li J , et al. The quality, safety and governance of telephone triage and advice services – an overview of evidence from systematic reviews. BMC Health Serv Res 2017; 17: 614. 28854916 10.1186/s12913-017-2564-x PMC5577663

[9] Sexton V Dale J Bryce C , et al. Service use, clinical outcomes and user experience associated with urgent care services that use telephone-based digital triage: a systematic review. BMJ Open 2022; 12: e051569. 10.1136/bmjopen-2021-051569 PMC8724705 34980613

[10] Chambers D Cantrell AJ Johnson M , et al. Digital and online symptom checkers and health assessment/triage services for urgent health problems: systematic review. BMJ Open 2019; 9: e027743. 10.1136/bmjopen-2018-027743 PMC6688675 31375610

[11] Blank L Coster J O’Cathain A , et al. The appropriateness of, and compliance with, telephone triage decisions: a systematic review and narrative synthesis. J Adv Nurs 2012; 68: 2610–2621. 22676805 10.1111/j.1365-2648.2012.06052.x

[12] Tran DT Gibson A Randall D , et al. Compliance with telephone triage advice among adults aged 45 years and older: an Australian data linkage study. BMC Health Serv Res 2017; 17: 512. 28764695 10.1186/s12913-017-2458-y PMC5539620

[13] Nakubulwa MA Greenfield G Pizzo E , et al. To what extent do callers follow the advice given by a non-emergency medical helpline (NHS 111): a retrospective cohort study. PLOS One 2022; 17: e0267052. 35446886 10.1371/journal.pone.0267052 PMC9022858

[14] Pope C Turnbull J Jones J , et al. Has the NHS 111 urgent care telephone service been a success? Case study and secondary data analysis in England. BMJ Open 2017; 7: e014815. 10.1136/bmjopen-2016-014815 PMC5623427 28576895

[15] Mansab F Bhatti S Goyal D . Reliability of COVID-19 symptom checkers as national triage tools: an international case comparison study. BMJ Health Care Inf 2021; 28: e100448. 10.1136/bmjhci-2021-100448 PMC8523957 34663637

[16] Lai L Wittbold KA Dadabhoy FZ , et al. Digital triage: novel strategies for population health management in response to the COVID-19 pandemic. Healthcare 2020; 8: 100493. 33129176 10.1016/j.hjdsi.2020.100493 PMC7586929

[17] Pope C Prichard J Turnbull J , et al. Ethnographic study of patient pathways and workforce implications of NHS 111 Online. England: National Institute for Health and Care Research, 2019.

[18] Devers KJ . How will we know “good” qualitative research when we see it? Beginning the dialogue in health services research. Health Serv Res 1999;34: 1153–1188. 10591278 PMC1089058

[19] Fox NJ . Post-positivism. In: Given LM (ed.) The SAGE encyclopedia of qualitative research methods. 2. London, England, UK: Sage, 2008, pp. 659–664.

[20] Tong A Sainsbury P Craig J . Consolidated criteria for reporting qualitative research (COREQ): a 32-item checklist for interviews and focus groups. Int J Qual Health Care 2007; 19: 349–357. 17872937 10.1093/intqhc/mzm042

[21] The Royal Australian College of General Practitioners. General Practice: Health of the Nation 2020. East Melbourne, VIC: RACGP, 2020.

[22] Healthdirect Australia. Our history 2019 [Available from: https://about.healthdirect.gov.au/our-history.

[23] Clay-Williams R Baysari M Taylor N , et al. Service provider perceptions of transitioning from audio to video capability in a telehealth system: a qualitative evaluation. BMC Health Serv Res 2017; 17: 1–8. 28806903 10.1186/s12913-017-2514-7 PMC5557607

[24] Otter.ai. Otter.ai. Los Altos, CA, US: Otter.ai, 2016.

[25] Kiger ME Varpio L . Thematic analysis of qualitative data: AMEE guide No. 131. Med Teach 2020; 42: 846–854. 32356468 10.1080/0142159X.2020.1755030

[26] Braun V Clarke V . Using thematic analysis in psychology. Qual Res Psychol 2006; 3: 77–101.

[27] Swerissen H . Toward greater integration of the health system. Aust Health Rev 2002; 25: 88–93. 12474504 10.1071/ah020088

[28] Freeman T Baum F Javanparast S , et al. Challenges facing primary health care in federated government systems: implementation of primary health networks in Australian states and territories. Health Policy 2021; 125: 495–503. 33602531 10.1016/j.healthpol.2021.02.002

[29] Baum F Ziersch A Freeman T , et al. Strife of interests: constraints on integrated and co-ordinated comprehensive PHC in Australia. Soc Sci Med 2020; 248: 112824. 32058888 10.1016/j.socscimed.2020.112824

[30] Javanparast S Baum F Freeman T , et al. Collaborative population health planning between Australian primary health care organisations and local government: lost opportunity. Aust N Z J Public Health 2019; 43: 68–74. 30296822 10.1111/1753-6405.12834

[31] Kujala S Hörhammer I Hänninen-Ervasti R , et al. Health professionals’ experiences of the benefits and challenges of online symptom checkers. Stud Health Technol Inform 2020; 270: 966–970. 32570525 10.3233/SHTI200305

[32] Kujala S Hörhammer I Kaipio J , et al. Health professionals’ expectations of a national patient portal for self-management. Int J Med Inf 2018; 117: 82–87. 10.1016/j.ijmedinf.2018.06.005 30032968

[33] healthdirect Australia. Annual Report – Financial Year 2019–2020. Haymarket, NSW, AU: healthdirect Australia, 2020.

[34] Pavlova A Witt K Scarth B , et al. The Use of Helplines and Telehealth Support in Aotearoa/New Zealand During COVID-19 Pandemic Control Measures: A Mixed-Methods Study. Front Psychiatry 2022; 12: 2604. 10.3389/fpsyt.2021.791209 PMC8833513 35153859

[35] Duckett S . What should primary care look like after the COVID-19 pandemic? Aust J Prim Health 2020; 26: 207–211. 32454003 10.1071/PY20095

[36] Knowles E O’Cathain A Turner J , et al. Effect of a national urgent care telephone triage service on population perceptions of urgent care provision: Controlled before and after study. BMJ Open 2016; 6: e0118846. 10.1136/bmjopen-2016-011846 PMC5073559 27742622

[37] Churruca K Pomare C Ellis LA , et al. The influence of complexity: a bibliometric analysis of complexity science in healthcare. BMJ Open 2019; 9: e027308. 10.1136/bmjopen-2018-027308 PMC6475366 30904877

